# Monoamine oxidase A activity in fibroblasts as a functional confirmation of *MAOA* variants

**DOI:** 10.1002/jmd2.12194

**Published:** 2020-12-28

**Authors:** Tessa M. A. Peters, Irma Lammerts van Bueren, Ben P.B.H. Geurtz, Karlien L. M. Coene, Nicole de Leeuw, Han G. Brunner, Jón J. Jónsson, Michèl A. A. P. Willemsen, Ron A. Wevers, Marcel M. Verbeek

**Affiliations:** ^1^ Department of Neurology Donders Institute for Brain, Cognition and Behavior, Radboud University Medical Center Nijmegen The Netherlands; ^2^ Department of Laboratory Medicine, Translational Metabolic Laboratory (TML) Radboud University Medical Center Nijmegen The Netherlands; ^3^ Department of Human Genetics and Donders Centre for Cognitive Neuroscience Radboud University Medical Center Nijmegen The Netherlands; ^4^ Department of Clinical Genetics Maastricht University Medical Center+ Maastricht The Netherlands; ^5^ Department of Genetics and Cell Biology Maastricht University Medical Center+ Maastricht The Netherlands; ^6^ GROW Institute for Developmental Biology and Cancer Maastricht University Medical Centre Maastricht The Netherlands; ^7^ Department of Genetics and Molecular Medicine Landspitali University Hospital Reykjavik Iceland; ^8^ Department of Biochemistry and Molecular Biology, Faculty of Medicine University of Iceland Reykjavik Iceland; ^9^ Department of Pediatric Neurology Donders Institute for Brain, Cognition and Behavior, Radboud University Medical Center Nijmegen The Netherlands

**Keywords:** enzyme assay, functional confirmation, HPLC, MAO‐A deficiency, variant of uncertain significance

## Abstract

Monoamine oxidase A (MAO‐A) deficiency is a rare inborn error of metabolism with impaired degradation of biogenic amines including 5‐hydroxytryptamine (5‐HT), resulting in borderline intellectual disability and behavioral abnormalities. Genetic variants in *MAOA* need functional confirmation to enable a definite diagnosis. To this end, we developed an inexpensive, simple and nonradioactive MAO‐A activity assay based on the conversion of 5‐HT into 5‐hydroxyindoleacetic acid (5‐HIAA). Fibroblast cell lysates were incubated with 5‐HT and aldehyde dehydrogenase to allow 5‐HIAA production. 5‐HIAA was quantified using high‐performance liquid chromatography with fluorimetric detection. We optimized reaction mixture components, pH, and substrate concentration and tested linearity and specificity of the assay. We verified the functional validity of the enzyme assay using fibroblasts of controls, female mutation carriers and MAO‐A deficient patients. This included a newly described patient with a novel *MAOA* variant (c.1336G>A, p.(Glu446Lys)), who represents the fifth MAO‐A deficiency family so far. The optimized enzyme assay showed good linearity and specificity. Application to clinical samples showed a 100% differentiation of affected patients (with negligible MAO‐A enzyme activity) and controls or mutation carriers. In conclusion, the described MAO‐A activity assay is easy to implement and can readily be used to test the pathogenicity of variants in the *MAOA* gene in a clinical setting. Especially in this era of whole‐exome (and whole‐genome) sequencing, this functional assay fulfills a clinical need for functional confirmation of a suspected diagnosis of MAO‐A deficiency.

**Synopsis**

Our inexpensive, simple and nonradioactive MAO‐A activity assay in fibroblasts provides functional confirmation of the pathogenicity of *MAOA* variants in previously known patients as well as in a newly described patient.

## 1 INTRODUCTION

Monoamine oxidase A (MAO‐A) is an enzyme with a pivotal role in neurotransmitter metabolism. It is responsible for the deamination of several biogenic amines including serotonin (5‐hydroxytryptamine, 5‐HT), norepinephrine, and dopamine. Deficiency of MAO‐A was first described in a Dutch family more than 25 years ago.^1^ The disease, also known as Brunner syndrome (OMIM #300615), results from a defect in the *MAOA* gene, located on the X‐chromosome (Xp11.3).^2^ Affected males in this family presented with borderline intellectual disability and abnormal behavior including impulsive aggression. Since then, only three more families with MAO‐A deficiency have been reported.^3^, ^4^, ^5^ Biochemically, these cases are all characterized by an abnormal urinary monoamine excretion pattern which includes elevated normetanephrine, metanephrine, and 5‐HT.^5^ Of note, in one of the families, the metabolite levels were completely normalized by treatment with a selective serotonin reuptake inhibitor, emphasizing the importance of an accurate diagnosis.

While 5‐HT and norepinephrine are specific substrates for MAO‐A, dopamine is also degraded by the isoform, MAO‐B. Next to that, MAO‐B specifically catalyzes the deamination of benzylamine and phenethylamine, making the functions of the two isoforms only partially overlapping. In many different tissues, including brain, liver, and kidney, both enzymes are present. However, the placenta specifically expresses MAO‐A and blood platelets only express MAO‐B.^6^ MAO‐B deficiency (OMIM #309860) has also been described, with patients who do not show any behavioral symptoms.^7^ Furthermore, as the *MAOA* and *MAOB* genes are located close together, a combined deficiency of both enzymes also exists.^7^ For the functional confirmation of a genetic diagnosis of MAO‐B deficiency, the activity in blood platelets can be measured.^8^ Especially when a variant of unknown significance (VUS) is found, this is an essential step. However, as noted, platelets do not contain MAO‐A. Moreover, changes in bioamine metabolism in urine, serum, or plasma can be indicative of disturbances of MAO‐A activity but are not always consistent between different patients.^5^ As a result, MAO‐A deficiency needs to be functionally confirmed by measuring the enzyme activity in cells that do express MAO‐A.

**FIGURE 1:**
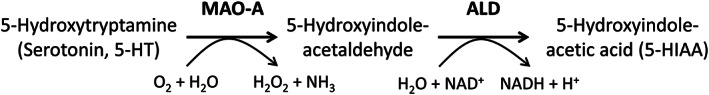
The formation of 5‐hydroxyindoleacetic acid from 5‐hydroxytryptamine. 5‐Hydroxytryptamine (5‐HT) is first converted to 5‐hydroxyindoleacetaldehyde by monoamine oxidase A (MAO‐A). This is then further oxidized by aldehyde dehydrogenase (ALD) to 5‐hydroxyindoleacetic acid (5‐HIAA). NAD^+^: nicotinamide adenine dinucleotide (oxidized); NADH: nicotinamide adenine dinucleotide (reduced)

An assay for MAO‐A activity in fibroblasts has previously been described in the literature.^9^ This is, however, a radiometric assay, which requires radioactively labeled metabolites and does therefore not fit into the standard routine of a metabolic laboratory. Our aim was to develop an inexpensive, simple and nonradioactive MAO‐A activity assay. Since 5‐hydroxyindoleacetic acid (5‐HIAA) is routinely detected in urine for neurometabolic diagnostics and requires MAO‐A for its production (see Figure 1), we based our assay on measurement of this metabolite.

In this article, we describe an assay for MAO‐A activity in fibroblasts based on measurement of 5‐HIAA using high‐performance liquid chromatography (HPLC) with fluorimetric detection. We have tested the linearity and specificity of the assay and verified its functionality using fibroblasts of controls, mutation carriers and MAO‐A deficient patients.

## 2 METHODS

### 2.1 Chemicals and reagents

Milli‐Q water was prepared using the Milli‐Q Advantage A10 from Merck‐Millipore (Burlington, MA). Potassium‐activated aldehyde dehydrogenase (ALD) from baker's yeast (*S. cerevisiae*) was purchased from Merck KGaA (Darmstadt, Germany) as a lyophilized powder with ≥2.0 units/mg protein (product number A6338). Ammonium acetate, citric acid, clorgyline (product number M3778), deprenyl (product number S036000), DL‐dithiothreitol (DTT), flavin adenine dinucleotide (FAD), 5‐HIAA, 5‐HT, hydrogen chloride, methanol, nicotinamide, β‐nicotinamide adenine dinucleotide hydrate (β‐NAD), perchloric acid (PCA), and potassium phosphate were all purchased from Merck KGaA as well.

### 2.2 Subjects

Fibroblasts were obtained from 10 controls, 2 female mutation carriers, and 4 affected patients. Their characteristics are summarized in Table 1. Control fibroblasts were randomly selected from cell lines cultured at our metabolic laboratory (Translational Metabolic Laboratory, Laboratory Medicine, Radboudumc) for which consent for the use in assay validation was present and processed anonymously. These originated from 3 mm punch biopsies of the skin.

**TABLE 1:** Subject characteristics

	Control^a^	Carrier	Affected
N	10	2	4
Sex (F/M)	4/6	2/0	0/4
Age at biopsy (y)	12.1 (mean) ± 15.4 (sd)	38.5, 47.6	34.5 (mean) ± 17.3 (sd)
*MAOA* genotype (transcript NM_000240.3)	wt(/wt)	c.886C>T, p.Gln296Ter/wt	c.886C>T, p.Gln296Ter (n = 3); c.1336G> A, p.(Glu446Lys) (n = 1)

^a^ Includes the unaffected father of one of the patients.

Abbreviation: sd, standard deviation.

The index patient of family 1 is a 7‐year‐old boy from Iceland who was referred for genetic evaluation because of temper tantrums, yelling, and antisocial tendencies, that is, violence and damaging property. His birth was induced at 37 weeks because of pre‐eclampsia. He presented a delay in reaching developmental milestones, including reactive smiling and eye tracking at 3 months, sitting at 9 months, and walking at 18 months. Physical examination showed frontal bossing, an occipital frontal circumference at the 95th percentile, slight hypotonia and a small hemangioma in the hair scalp. At 6 years of age, IQ evaluation showed borderline intellectual disability (IQ of 69). Over the years, the patient has received multiple medications including atomoxetine, methylphenidate, amitriptyline and melatonin. Whole‐exome sequencing at Centogene (Rostock, Germany) revealed a *de novo* mutation in *MAOA*: c.1336G>A, p.(Glu446Lys), in accordance with the negative family history. This previously undescribed variant lies in a conserved region and was classified as likely pathogenic (Polyphen probably damaging, SIFT deleterious, and Mutation Taster disease causing). Metabolic screening in urine showed a normal excretion pattern that excluded most organic acidurias and defects in the metabolism of creatinine, oligosaccharides and purines/pyrimidines. Neurotransmitter analysis showed increased serotonin in serum and increased serotonin, norepinephrine, epinephrine, dopamine, metanephrine, and normetanephrine in urine (see Table S1). The patient's fibroblasts were collected by punch biopsy. Fibroblasts of his unaffected father were collected as well and included as a sample in the control group.

The remaining patients and both carriers originated from the same family (family 2 in this article) and have previously been described as BB, AW and AX, and AY and AZ, respectively.^2^ The patients showed mild intellectual disability and abnormal behavior, in particular aggression, while the female carriers did not show any symptoms. All family members previously signed a general informed consent for the use of their fibroblasts in research, after which the skin fibroblasts were collected using punch biopsies.

### 2.3 Cell culture

Fibroblasts from random controls and family 1 were cultured in M199 medium (Pan Biotech, Aidenbach, Germany) supplemented with 10% v/v fetal calf serum (FCS; Gibco/Life Technologies, Grand Island, NY) and 1% v/v penicillin/streptomycin (pen/strep; Gibco/Life Technologies) in a humidified atmosphere with 5% CO_2_ at 37°C. Fibroblasts from family 2 were cultured in DMEM with 20% FCS, 1% pen/strep, and 1% sodium pyruvate in a humidified atmosphere with 7.5% CO_2_ at 37°C (all reagents from Sigma‐Aldrich). Fibroblast pellets were washed with PBS three times before freezing at −80°C.

### 2.4 Measurement of MAO‐A activity

Fibroblasts were thawed and subsequently lysed by resuspension in Milli‐Q at ~10*10^6^ cells/100 μL followed by sonification on ice (3 × 10 seconds, 10%, Branson Digital Sonifier type W‐250D). To 100 μL cell lysate, we added 150 μL 267 mM potassium phosphate buffer pH 7.5 and 100 μL of a mix containing 6 U/mL ALD, 26 mM DTT and 22 mM β‐NAD. This was incubated in open glass tubes in a shaking water bath for 10 minutes at 37°C. For the specificity tests, the incubation was prolonged to 30 minutes and 10 μL clorgyline (MAO‐A inhibitor^10^) or deprenyl (MAO‐B inhibitor^11^) was added to the mixture using different concentrations. Based on previous literature stating that both clorgyline and deprenyl effectively blocked MAO‐A and MAO‐B, respectively, at concentrations of 1 μM,^12^ we tested inhibitor concentrations ranging from 10^−4^ to 10^2^ μM.

After preincubation, the reaction mixture was complemented with 5‐HT solution (or MilliQ in case of the blank) to obtain a final volume of 400 μL and a 5‐HT concentration of 300 μM. After 2 hours of incubation, the reaction was stopped using 200 μL 2 M citric acid. Protein was then precipitated by adding 100 μL 1 M PCA to 300 μL of the final reaction mixture and centrifugation (5 minutes, 20 238*g*). We then measured the 5‐HIAA concentration in the supernatant using HPLC (1525 Binary HPLC Pump and Atlantis dC18 Column, 100 Å, 5 μm, 4.6 mm × 150 mm; Waters, Milford, MA, USA) with fluorimetric detection (Yex = 280 nm, Yem = 325 nm, gain = 1000; FP‐2020 Intelligent Fluorescence Detector; Jasco, Hachioji, Tokyo, Japan). The mobile phase consisted of 1.54 g of ammonium acetate dissolved in 2 L of Milli‐Q and 100 mL methanol, which was titrated to pH 5.7 using hydrogen chloride. The injection volume was 100 μL, the run time 30 minutes, and the flow 1.25 mL/min. Depending on the height of the observed peak, if necessary, the measurement was repeated with a twofold, fivefold or 10‐fold dilution of the supernatant with 0.3% HCOOH to ensure accurate quantification.

For each sample, the concentration was corrected by subtracting the concentration of the blank for the same cell lysate. Furthermore, as a standard, 400 μL of 100 nM 5‐HIAA was also treated with citric acid and protein precipitation and subject to 5‐HIAA detection. Total protein was determined using the Lowry protein assay.^13^

Using a single control fibroblast cell line, several parameters were tested during the development of the method. First, enzyme kinetics were evaluated using a substrate range of 1 to 500 μM. For calculations of Vmax and Km, it was assumed that the conversion by MAO‐A (5‐HT to 5‐hydroxyindoleacetaldehyde) was the rate‐limiting step, as ALD (which converts 5‐hydroxyindoleacetaldehyde to 5‐HIAA) was present in excess amounts. Subsequently, the effect of the presence of 87.5 μM FAD or 0.5 μM nicotinamide in the reaction mixture was tested. The presence of ALD, DTT, and β‐NAD (at the described concentrations) was evaluated as well. The linearity of the assay was assessed over the range of 30 minutes to 4 hours. As mentioned, clorgyline (10^−4^ μM to 10^1^ M) and deprenyl (10^−3^ M to 10^2^ M) were added to test specificity and their half maximal inhibitory concentration (IC_50_) was calculated.

### 2.5 Statistics

All comparisons involving two groups were tested using the Wilcoxon signed‐rank test. Correlation was calculated using Spearman's rank correlation coefficient (ρ).

## 3 RESULTS

### 3.1 Optimization of the enzyme assay

The relation between different substrate (5‐HT) concentrations and 5‐HIAA production is shown in Figure S1A. Based on this, we chose a substrate concentration of 300 μM for the final assay. From the accompanying Lineweaver‐Burk plot (Figure S1B), we calculated the Km to be 50 μM 5‐HT and the Vmax 1.3*10^−4^ μmol/h.

We tested the effect of the presence of FAD, a cofactor covalently bound to MAO‐A, and nicotinamide, a precursor for the ALD cofactor NAD^+^. Neither addition of FAD nor of nicotinamide affected 5‐HIAA production, so both compounds were excluded from the reaction mixture. In contrast, the addition of 26 mM DTT (which reduces disulfide bonds in proteins) and 22 mM β‐NAD (an ALD co‐factor) resulted in a fivefold increased production of 5‐HIAA. When we added 6 U/mL ALD as well, the production of 5‐HIAA rose further to a sevenfold increase compared to the control incubation. Therefore, DTT, β‐NAD, and ALD were all included in the final protocol.

To optimize the pH for the reaction we first tested a broad range of pH values, which indicated that the optimal pH for the reaction was approximately pH 7‐8 (Figure S2A). Further narrowing down the range yielded pH 7.5 as the optimal pH. We used reaction times ranging from 0 to 4 hours to test the linearity of the reaction. Figure S2B shows that the reaction remained linear over the full tested range, including a reaction time of 2 hours, which was used in the final assay.

**FIGURE 2:**
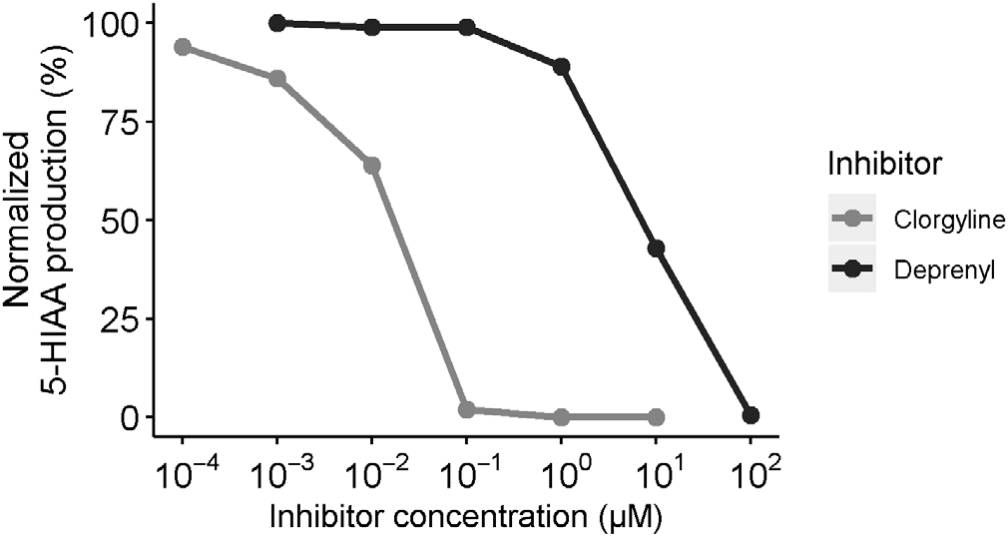
Effects of clorgyline (specific MAO‐A inhibitor) and deprenyl (specific MAO‐B inhibitor) on the 5‐HIAA production as a measure of MAO‐A activity. IC_50_(clorgyline) = 0.017 μM, IC_50_(deprenyl) = 7.0 μM

Clorgyline, a specific MAO‐A inhibitor, strongly decreased the MAO‐A activity as measured by the assay, with almost complete inhibition already at 0.1 μM (Figure 2). Deprenyl on the other hand, which is a specific inhibitor of MAO‐B, hardly affected the reaction until a concentration of 10 μM. A deprenyl concentration of 100 μM was necessary to completely eliminate MAO‐A activity. By interpolation, the IC_50_ of clorgyline was determined to be 0.017 μM, while the IC_50_ of deprenyl was 7.0 μM.

### 3.2 Application of the enzyme assay to clinical samples

**FIGURE 3:**
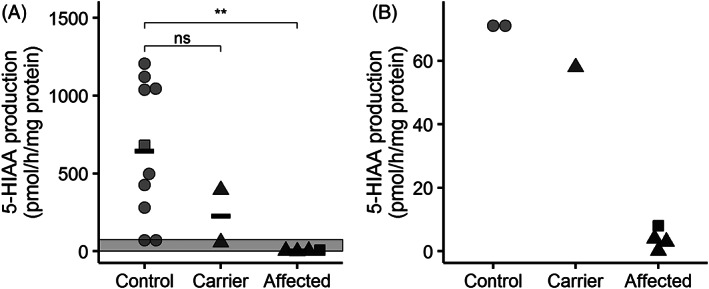
5‐HIAA production as a measure of MAO‐A activity in controls (n = 9), carriers (n = 2) and affected patients (n = 3). Circles represent random controls, squares belong to family 1, and triangles belong to family 2. Panel A shows the full range, panel B zooms in on the indicated area in A. Horizontal bars represent group means. ns: not significant (*P* > .05), ** *P* <= .01 (as calculated by Wilcoxon signed‐rank test)

The optimized assay conditions were applied to the fibroblast cell lines of 10 controls (of which six male), two female carriers and four affected male patients. MAO‐A enzyme activity (as measured by 5‐HIAA production) in controls covered a wide range (71‐1206 pmol/h/mg protein; Figure 3). We did not find a significant association with age (ρ = 0.62, *P* = .056) or sex (*P* = 1). Mutation carriers had an activity similar to the lower range of the controls (58‐394 pmol/h/mg protein; *P* = .16 compared to controls). In the affected MAO‐A deficient patients, enzyme activity was almost completely absent (0.2‐8.0 pmol/h/mg protein; *P* = .0058 compared to controls), showing a complete separation from carriers and controls. MAO‐A enzyme activity in the deficient patients was thus at least sevenfold lower than the lowest enzyme activity of 58 pmol/h/mg protein observed in controls and mutation carriers. For the patient of family 1, the low activity was a functional confirmation of the genetic diagnosis of MAO‐A deficiency.

## 4 DISCUSSION

We developed an assay to assess the activity of the MAO‐A enzyme in a clinical setting, using 5‐HIAA production in fibroblasts as quantified by HPLC with fluorimetric detection as an outcome measure. The assay showed a good linearity and specificity. Application to fibroblasts of controls, carriers, and patients affected by MAO‐A deficiency showed a 100% distinction between affected patients on the one hand and controls or mutation carriers on the other hand. As expected, the MAO‐A enzyme activity in patients was nearly undetectable. Therefore, this enzyme assay may serve as a functional confirmation for a suspected diagnosis of MAO‐A deficiency. The patient group included a previously undescribed isolated case of MAO‐A deficiency, representing the fifth family with this disorder described so far.

In controls, we observed a broad range of enzyme activity levels, which has been reported before.^14^, ^15^ Possibly, the activity is affected by polymorphisms within the *MAOA* gene itself or other genes involved in expression or posttranslational modification of the enzyme. For example, the 4‐repeat allele of the upstream variable number tandem repeat polymorphism (uVNTR) has been shown to significantly increase the MAO‐A activity compared to the 3‐repeat allele in male skin fibroblast cultures.^16^ Even more variation in activity could be attributed to a variable proportion (0.5‐90%) of fibroblasts actually expressing the MAO‐A enzyme.^16^, ^17^ Previous literature also identified a positive correlation between activity and age,^15^ which may be attributed to age‐dependent changes in the DNA controlling MAO‐A activity or in the proportion of fibroblasts that express MAO‐A. However, this was found by combining cohorts to a large total number of samples, which may explain why we could not reproduce this in our relatively small control group.

Despite the relatively low number of samples, we obtained a perfect separation of the enzyme activities of MAO‐A deficient patients from the other samples. Therefore, the large amount of variation in the control group did not hamper the interpretation of the assay results. As the carriers were in the lower range of our controls, they seemed to be unaffected in their activity. These findings are in accordance with earlier studies.^7^ The X‐inactivation pattern (determined at our center as described previously^18^) in the fibroblasts of the carrier with the lowest MAO‐A activity was skewed, with an 80% expression of the healthy allele. However, the carrier with the higher activity did not show a skewed X‐inactivation pattern. This indicates that even partial expression of *MAOA* can be sufficient for normal activity of the enzyme.

A disadvantage of the use of fibroblasts is that the cells need to be obtained by an invasive skin biopsy. From this perspective, applying the assay to blood cells would be preferred. However, the conversion of 5‐HT (ie, the MAO‐A activity) in these cells is quite low,^19^ impeding reliable quantification. Additionally, in pilot experiments where we applied our assay to plasma or serum rather than fibroblasts, we were not able to specifically detect 5‐HIAA. Epithelial cells from urine may also be used for functional testing,^20^ but culturing these cells only has a limited success rate, making them impractical for diagnostic purposes. We therefore conclude that fibroblasts are the optimal source for measuring MAO‐A activity in humans.

As noted, a major advantage of our assay is that radioactive compounds are not required. In addition, we used a substrate (5‐HT) that is specific for MAO‐A. This was confirmed by our specificity testing, which showed that the assay was effectively inhibited by the known MAO‐A inhibitor clorgyline and that the MAO‐B inhibitor deprenyl only had an effect when applied in the micromolar range. This is much higher than its reported inhibitory effect on MAO‐B, which is in the nanomolar range.^12^ Consequently, the use of deprenyl is not necessary in the final assay, as opposed to the previously used assay based on tryptamine.^9^ Of note, another assay that measures MAO‐A activity based on HPLC and fluorimetric detection of 5‐HIAA has been described.^21^ However, it was applied to rat brain tissue rather than human fibroblasts and it has not been validated in the context of MAO‐A deficiency. Commercial kits based on fluorimetric 5‐HIAA are available as well, but these are aimed at high‐throughput measurements and are not to be used for diagnostic purposes. Therefore, we believe our assay is unique and suitable for clinical application in case of a VUS in *MAOA* and/or a deviant monoamine pattern in body fluids suggestive of MAO‐A deficiency.

In conclusion, the described MAO‐A activity assay is easy to implement and can readily be used to test the pathogenicity of variants in the *MAOA* gene in a clinical setting. Especially in this era of whole‐exome (and whole‐genome) sequencing, this functional assay fulfills a clinical need for functional confirmation of a suspected diagnosis of MAO‐A deficiency.

## CONFLICT OF INTEREST

The authors declare no conflicts of interest.

## AUTHOR CONTRIBUTIONS

Tessa M. A. Peters: Interpreted the data and drafted the manuscript. Guarantor. Irma Lammerts van Bueren: Designed and executed the experiments, performed data analysis and interpretation, and revised the manuscript. Ben P.B.H. Geurtz: Designed and executed the experiments, performed data analysis and interpretation, and revised the manuscript. Karlien. L. M. Coene: Contributed to data interpretation and revised the manuscript. Nicole de Leeuw: Contributed to clinical data collection, experimental design and data interpretation, and revised the manuscript. Han G. Brunner: Contributed to clinical data collection, experimental design and data interpretation, and revised the manuscript. Jón J. Jónsson: Contributed to clinical data collection and revised the manuscript. Michèl A.A.P. Willemsen: Contributed to data interpretation and revised the manuscript. Ron A. Wevers: Contributed to data interpretation and revised the manuscript. Marcel M. Verbeek: Contributed to experimental design and data interpretation, and revised the manuscript.

## DATA AVAILABILITY

Data supporting the findings of this study are available as electronic supplementary material.

## ETHICS STATEMENT

The study was conducted in accordance with the Declaration of Helsinki. All patients (or their guardians) approved of the possible use of their left‐over samples for method validation purposes, in agreement with institutional and national legislation. No animal subjects were used in this study.

## Supporting information


**Appendix** **S1**: Supporting Information
