# Supplementary material for: Monoamine oxidase A activity in fibroblasts as a functional confirmation of MAOA variants
Source: JIMD Rep. 2020 Dec 28;58(1):114–21. doi: 10.1002/jmd2.12194 (PMC7932864; doi:10.1002/jmd2.12194)
Supplement: Supplementary file 1 — Appendix S1: Supporting Information [file JMD2-58-114-s001.pdf]

Supplementary material

Table S1. Neurotransmitter analysis results of the index patient of Family 1.

**Metabolite**		**Value**	**Reference range**	**Unit**
**Urine (24h)**	Serotonin	**521**	<200	µg/l
	Catecholamines	**141.8**	<115	µg/l
	Norepinephrine	**105.9**	<100	µg/l
	Epinephrine	**35.9**	<20	µg/l
	Dopamine	**644.8**	65-563	µg/l
	Metanephrine, normetanephrine	**2850**	<900	µg/l
	Normetanephrine	**2382**	<450	µg/l
	Metanephrine	**468.2**	<350	µg/l
**Serum**	Serotonin	**429**	101-283	µg/l

Bold-faced values are outside of the reference range.

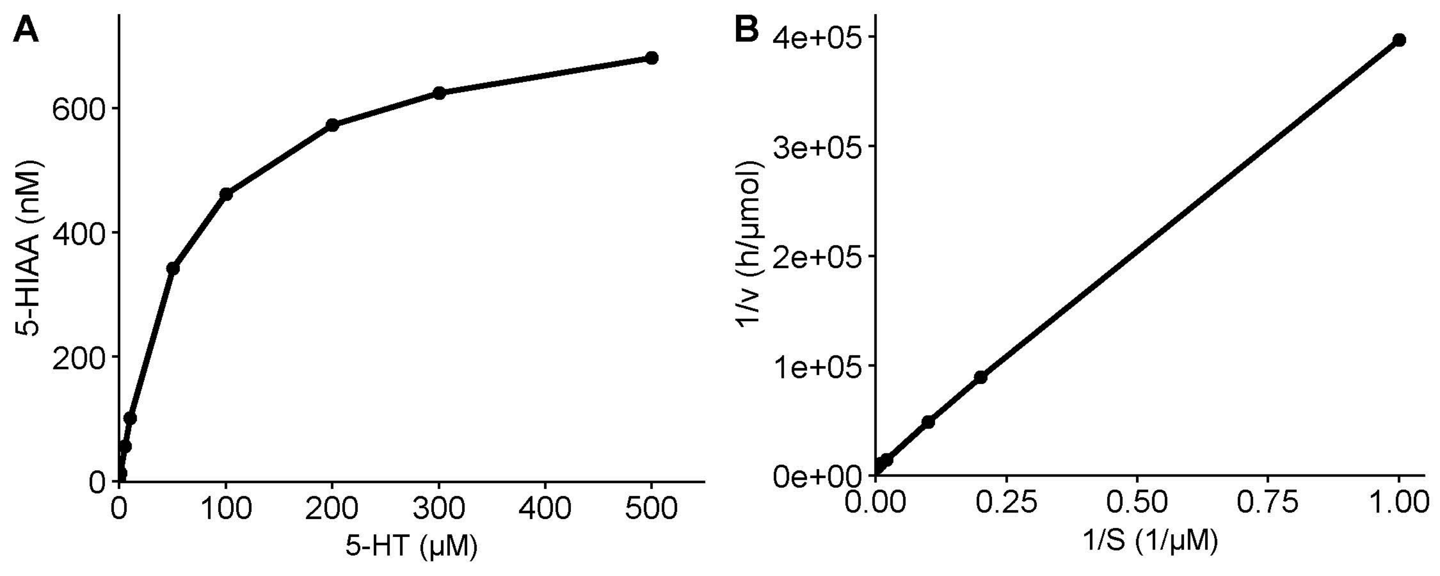

A
600
400
200
0
5-HIAA (nM)
0
100
200
300
400
500
5-HT (μM)
B
4e+05
3e+05
2e+05
1e+05
0e+00
1/v (h/μmol)
0.00
0.25
0.50
0.75
1.00
1/S (1/μM)

Figure S1. Substrate curve (A) and Lineweaver-Burk plot (B) for the MAO-A assay with reaction volume 400 µl and reaction time 2 h.

From B, it was calculated that Km = 50 µM and Vmax = 1.3*10-4 µmol/h. 5-HIAA = 5-hydroxyindoleacetic acid, 5-HT = 5-hydroxytryptamine, S = substrate, v = velocity.

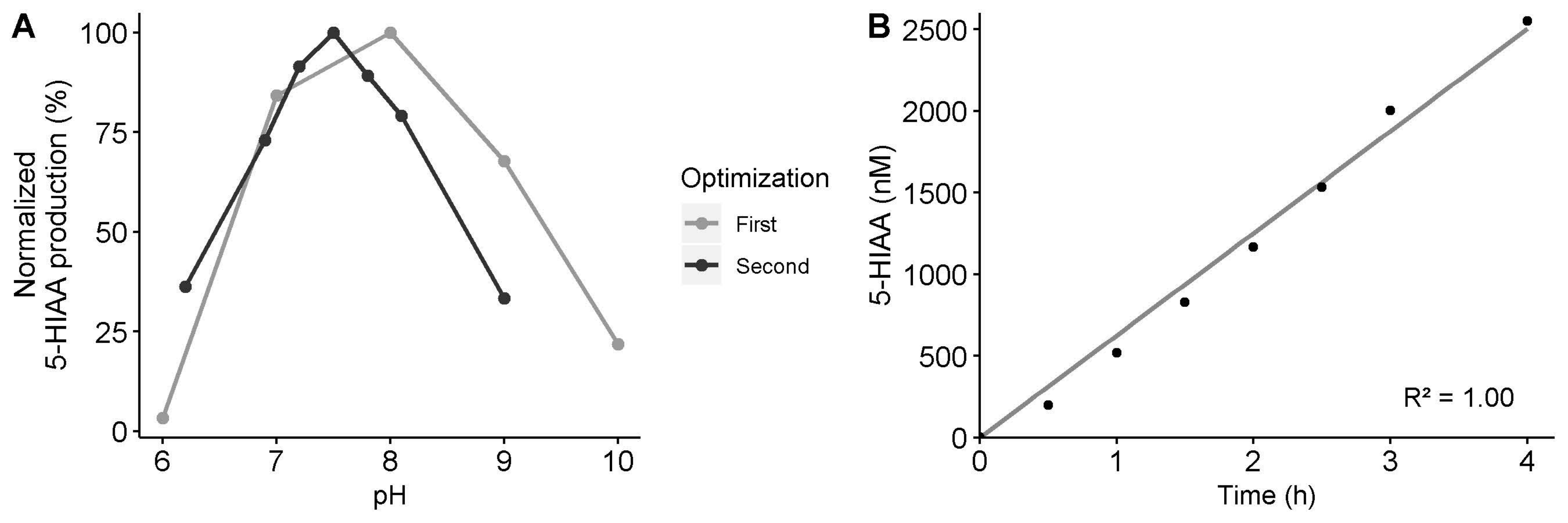
A
Normalized
5-HIAA production (%)
100
75
50
25
0
6
7
8
9
10
pH
Optimization
First
Second
B
5-HIAA (nM)
2500
2000
1500
1000
500
0
0
1
2
3
4
Time (h)
R² = 1.00

Figure S2. (A) pH curves for determination of the optimal pH for the used MAO-A assay. (B) 5-HIAA production with reaction times ranging from 0 to 4 h.

(A) A broad spectrum analysis of the effect of pH showed that the optimum was around 7 to 8 (first optimization), which was further tested using a narrower range with more measuring points (second optimization). Based on this, the optimal pH for the assay was determined to be 7.5. (B) The trendline and R^2 were calculated by linear regression with (0,0) as a set point.
